# Editorial: Metabolic dysfunction-associated fatty liver disease (MAFLD): innovative management strategies using herbal medicines

**DOI:** 10.3389/fphar.2025.1742956

**Published:** 2026-01-09

**Authors:** Yunhui Chen, Yujie Liu, Qing Zhang, Wei Peng

**Affiliations:** 1 CDUTCM-KEELE Joint Health and Medical Sciences Institute, School of Basic Medical Sciences, School of Modern Chinese Medicine Industry/School of Pharmacy, Chengdu University of Traditional Chinese Medicine, Chengdu, China; 2 Shanxi University of Chinese Medicine, Taiyuan, China; 3 Department of Molecular & Integrative Physiology, University of Michigan, Ann Arbor, MI, United States

**Keywords:** AMPK, herbal medicines, MAFLD, metabolic dysfunction-associated fatty liver disease, NAFLD, non-alcoholic fatty liver disease

Metabolic dysfunction-associated fatty liver disease (MAFLD), also known as non-alcoholic fatty liver disease (NAFLD), constitutes a substantial and growing global health burden, affecting approximately one-third of the adult population (https://doi:10.1136/gutjnl-2023-330595). Despite its high prevalence and progressive nature, current therapeutic strategies remain markedly limited, highlighting a critical demand for innovative treatment approaches. Within this context, herbal medicines have emerged as a promising direction for exploration. These natural interventions benefit from centuries of well-documented traditional application and exhibit multi-target therapeutic potential that aligns with the complex pathophysiology of MAFLD. This Research Topic, “Metabolic dysfunction-associated fatty liver disease (MAFLD): Innovative Management Strategies using Herbal Medicines,” presents ten high-quality research articles and reviews that systematically investigate how botanical preparations and natural metabolites influence crucial disease mechanisms. These encompass the AMP-activated protein kinase (AMPK) signaling pathway, autophagy regulation, pyroptosis, ferroptosis, and gut-liver axis communication, thereby collectively advancing our understanding of herbal interventions and inspiring innovative strategies for MAFLD management.

The original research article by Lv et al. examined the hepatoprotective effects of total flavones from Abelmoschus manihot (TFA) using a high-fat diet-induced MAFLD mouse model. Through integrated transcriptomic and metabolomic analytical approaches, the authors demonstrated that TFA administration significantly ameliorated hepatic steatosis, inflammatory response, and oxidative stress. Their research further identified that the underlying mechanism involved autophagy enhancement via inhibition of the PI3K/AKT/mTOR signaling pathway. In another experimental study, Yu et al. explored the therapeutic potential of a purified polysaccharide (BSP-1) from Bletilla striata in both *in vivo* and *in vitro* MAFLD models. Their results revealed that BSP-1 markedly improved serum lipid profiles and liver histopathology and exerted protective effects by suppressing the activation of the NLRP3/caspase-1/GSDMD pathway, consequently attenuating pyroptosis and hepatic inflammation. In addition, Chen et al. focused on quercetin, a bioactive compound isolated from *Zanthoxylum bungeanum* Maxim. Utilizing a combination of lipidomics and transcriptomics, their work elucidated that quercetin alleviated hepatic lipid accumulation and cellular damage by modulating the glycerophospholipid metabolism pathway and, significantly, inhibited ferroptosis through the p38 MAPK/ERK signaling axis.

Further, this Research Topic incorporates a series of insightful and timely reviews that systematically outline and interconnect emerging mechanistic frameworks and therapeutic strategies for MAFLD. Hao et al. comprehensively summarized how flavonoids modulate the gut-liver axis to ameliorate MAFLD, emphasizing multi-target regulation of PPARs, Nrf2, NF-κB, and FXR signaling, alongside innovative delivery systems and individualized nutritional strategies. Building upon the gut-liver dialogue, Zhang et al. elaborated on how botanical drugs and their metabolites target gut microbiota and hepatic immune responses, establishing a foundation for employing natural adjuvants to rectify immune dysfunction in MAFLD. Wen et al. highlighted the crosstalk between gut microbiota and mitochondrial function in obesity, elucidating how traditional Chinese medicine formulas and botanical metabolites may restore metabolic homeostasis by regulating this bidirectional axis. Wang et al. systematically reviewed natural active botanical metabolites that activate the AMPK signaling pathway, a central regulator of cellular energy, and provided a mechanistic foundation for their efficacy in improving lipid metabolic abnormalities in MAFLD. Zooming into specific botanicals, Xiao et al. illustrated the multi-target, multi-pathway mode of action of ginseng and its functional components, including saponins and non-saponins, in addressing NAFLD/MAFLD by regulating lipid metabolism, inflammation, and gut flora. Similarly, Chen et al. focused on *Scutellaria baicalensis* Georgi, detailing how its flavonoids exert anti-inflammatory, antioxidant, and lipid-regulating effects, thereby positioning it as a promising candidate for MAFLD intervention. Concluding this thematic thread, another review by Zhang et al. synthesized evidence on how natural products ameliorate NAFLD by targeting gut microbiota and lipid metabolism, underscoring their multi-target advantage in reducing hepatic steatosis and inflammation.

The findings and evidence yielded in this Research Topic underscore the therapeutic potential of herbal medicines in managing MAFLD through multi-target mechanisms, including AMPK activation, autophagy induction, pyroptosis and ferroptosis inhibition, and gut-liver-immune modulation. Nevertheless, certain aspects require more thorough investigation. Future studies should prioritize the conversion of preclinical efficacy into clinical validation via rigorously designed trials, intensify the exploration of synergistic effects among herbal components, and systematically elucidate the dose-response relationships and pharmacokinetic profiles of crucial bioactive metabolites. Moreover, the incorporation of emerging technologies, such as spatial multi-omics, single-cell sequencing, artificial intelligence, and organoid-based disease modeling, will further accelerate the interpretation of herb-host interactions and facilitate the development of standardized, evidence-based herbal formulations. By addressing these challenges, the field may effectively bridge traditional knowledge with modern precision medicine, paving the way for innovative, safe, and effective herbal-based interventions for MAFLD.

